# Thermal injury induces early blood vessel occlusion in a porcine model of brass comb burn

**DOI:** 10.1038/s41598-021-91874-0

**Published:** 2021-06-14

**Authors:** Jing Wang, Cheng Z. Wang, John R. Salsbury, Jianzi Zhang, Perenlei Enkhbaatar, David N. Herndon, Amina El Ayadi, Naseem H. Ansari

**Affiliations:** 1 Department of Biochemistry and Molecular Biology, University of Texas Medical Branch, 301 University Boulevard, Galveston, TX 77555-0647 USA; 2 Department of Surgery, University of Texas Medical Branch, 301 University Boulevard, Galveston, TX 77555 USA; 3 Department of Anesthesiology, University of Texas Medical Branch, Galveston, TX USA; 4 School of Health Professions, University of Texas Medical Branch, Galveston, TX USA

**Keywords:** Biochemistry, Cell biology, Molecular biology, Medical research

## Abstract

Burn wound progression is an important determinant of patient morbidity and mortality after injury. In this study, we used the brass comb contact burn to determine burn wound vertical injury progression with a focus on blood vessel occlusion and endothelial cell death. Class A 3-month-old Yorkshire pigs received a brass comb contact burn. Burn wounds were sampled at 0, 30 min, 1, 2, 4, and 24 h. Hematoxylin Phloxin Saffron staining and vimentin immunostaining were performed to determine the depth of blood vessel occlusion and endothelial cell death, respectively. The depth of blood vessel occlusion increased by 30 min (p < 0.005) and peaked by 1 to 4 h (p > 0.05). The depth of endothelial cell death risen to a plateau at 30 min (p < 0.005) to 2 h and then peaked at 24 h (p < 0.03). We observed a progression of blood vessel occlusion and vascular endothelial cell death from the middle of the dermis to the hypodermis within 2 h to 4 h after the initial injury, namely a progression from a second-degree (partial thickness) to third-degree (full thickness) burn. These data suggest that therapeutic interventions during this time window may provide a better outcome by reducing or preventing vertical progression of blood vascular occlusion or endothelial cell death.

## Introduction

Burn injury can be disastrous to the victims, their families, and society. In the United States, approximately 450,000 people required medical treatment according to the American Burn Association for burn-related injuries^1^, which costs about $18 billion on specialized care of these patients^2^.

Burn wound progression or burn conversion has been studied over half a century since Jackson described three zones of typical burn wounds (a central zone [zone of coagulation], the intermediate zone [zone of stasis], and the outer zone [zone of hyperemia]). The zone of necrosis does not initially undergo necrosis but experiences complete cessation of blood flow within the first 24 hours^3^. Since then, it has been recognized burn wounds undergo a dynamic progression during the first few days after injury. Blood flow cessation is one of the most important pathophysiological events that occur after burn regardless of wound infection status^3–8^.

Various experimental animal models were used to investigate the mechanisms underlying burn wound progression, with a focus on therapeutic interventions to minimize or prevent burn wound progression^1,4,9–19^. However, current clinical therapies do not include approved therapies that may limit or prevent^20^ burn wound progression^21^ warranting the need for more research in this area.

Failure to prevent burn wound progression may be due to the inappropriate timing of therapeutic interventions given the unclear definition of the time course of burn progression. While N-acetylcysteine was given (i.p.) at 1 h after burn^20^, r-tissue plasminogen activator was administrated (i.v.) at 2 h after injury^14^, erythropoietin was injected (i.p.) at 6 h after initial burn^22^ and the anti-TNF-α-HA-conjugates and antibodies to IL-6 were topically applied at 24 h after injury^23^. If a pathology component such as blood vessel occlusion is established by 2 h after burn, an intervention starting after 2 h will not prevent the pathology development.

Blood flow cessation at 24 h after injury, as Jackson presented initially^3^, is an important event that occurs during early burn wound progression. Decreased blood flow may cause the death of epithelial, endothelial, and mesenchymal cells, collagen degeneration, and tissue necrosis in the burn wounds thereafter. The importance of blood flow in burn progression evoked investigations to understand the time course and mechanisms of blood flow alterations after burn. Studies using postmortem arteriography of radiopaque dye to investigate dermal ischemia found that the wound circulation immediately after the burn was patent and unchanged from unburned controls^9^. However, the vascular compromise in the same study progressed to complete de-vascularization over 24 h, with the first evidence of blood vessel disruption between 2 and 4 h after injury^9^. Regas and Ehrlich^11^ used a laser Doppler flowmeter to monitor blood flow near the surface of normal control skin, burn sites, and unburned interspace in a live rat burn model with a brass comb burn. They revealed that the surface blood flow in unburned interspaces decreased by 73% at 24 h after injury. Blood flow in the burn sites was decreased by 83% of control values at the same time point^11^. Using a similar brass comb model, blood flow in the untreated interspace progressively declined to 25% of control at 5 min, and 10% of control from 1 to 4 h after injury^12,13^. Blood flow in the burn sites, however, dropped to 8% of control at 5 min after burn before increasing to 15% at 1 h and remained at the same level until 24 h after burn, suggesting that there is no progressive decline between 5 min and 24 h in the burn sites (zone of coagulation). Subsurface dermal blood flux, measured by Doppler flowmetry and fiber-optic confocal, exhibited a significant and progressive decrease in the zone of coagulation every hour during a 4-h study period when compared to corresponding control non-burned animals^15^. These studies warrant the need for a comprehensive analysis of the early vascular response to burn. While histological alterations of burn wounds are well documented^11–13,24–26^, few studies are focusing on early morphological blood vessel response to thermal injury. The current study was designed to provide a morphometric time course of blood vessel response at an early time point (30 min after injury) and shorter time intervals (30 min, 1, 2, 4, and 24 h) in a porcine brass comb model of burn by measuring the depth of blood vessel occlusion and endothelial cell death. Burn wound progression has two directions: horizontal and vertical. In burn patients, a large area of the burn site (zone of coagulation) is usually surrounded by a very narrow or small strip of stasis and hyperemia. Vertical injury progression in burn sites is more relevant to clinical practice since it is the mechanism for superficial second-degree burn wounds to progress to deep second-degree, or third-degree (full) burn wounds. Thus, the current study will focus on the vertical progression of blood vessel occlusion and endothelial cell death in the burn sites.

## Results

### Blood vessel occlusion

#### Histochemistry features

**Figure 1:**
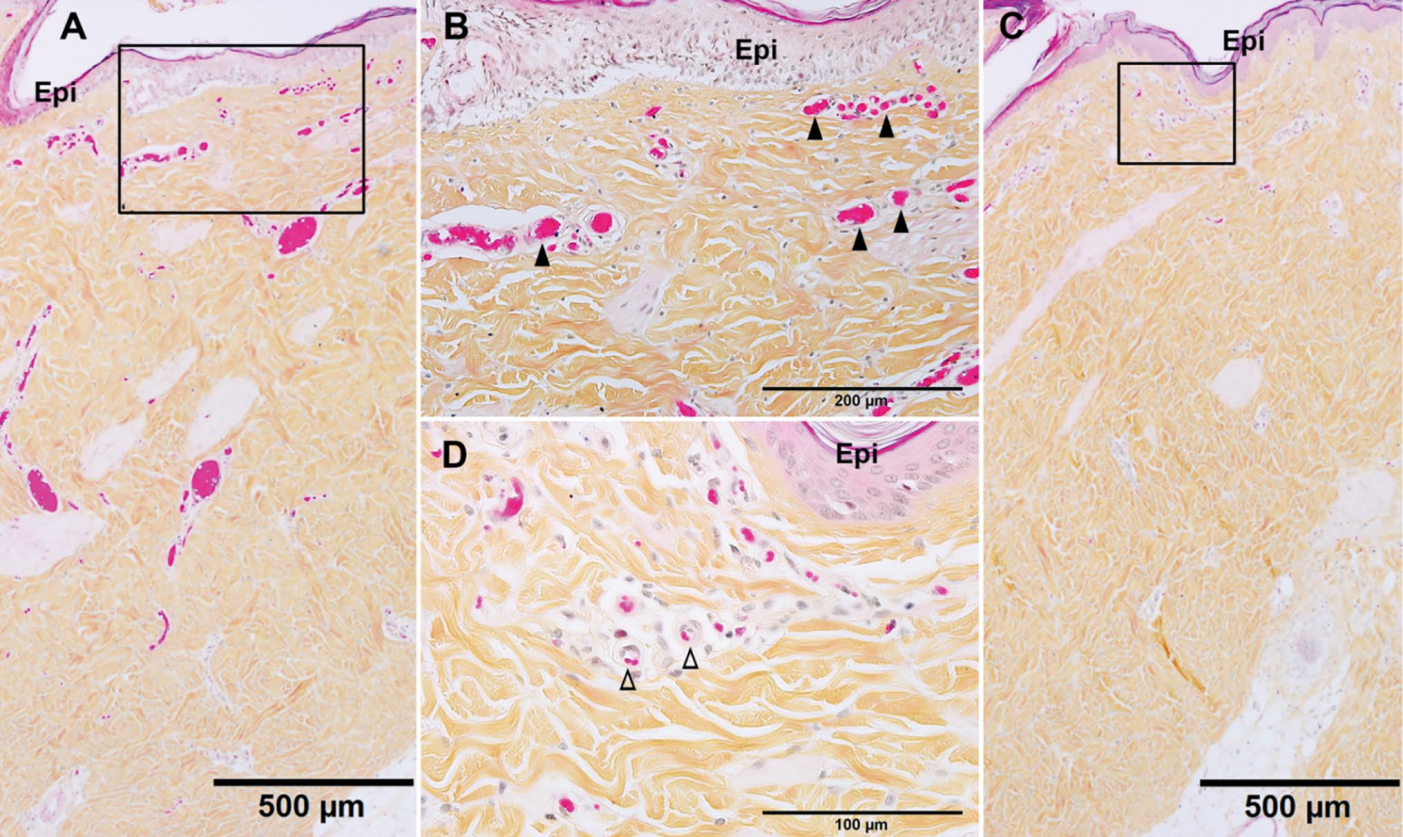
Representative HPS micrographs of burn site (**A,B**) and interspace (**C,D**) of a porcine burn wound 30 min after burn. (**B**) and (**D**) are higher power of the boxes in A and C, respectively. ▲, blocked small vessels beneath the epidermis. ∆, Clear vessels beneath the epidermis of interspace. Bar = 500 µm (**A,C**), 200 µm (**B**), 100 µm (**D**).

Successful 5-step Hematoxylin phloxin saffron (HPS) staining differentiates the most common connective tissue (collagen) as yellow, muscle, and cytoplasm including red blood cells both as pink, and cell nuclei as blue (Fig. 1). At 30 min after brass comb contact burn (the earliest time point examined in this study), blood vessel occlusion was observed just beneath the epidermis on the burn sites and was featured with dilated venules and/or arterioles filled with erythrocyte aggregates and/or denatured clots (Fig. 1A, B). The uninjured interspaces, however, showed clear blood vessels in the dermis near the epidermis (Fig. 5C, D).

#### Progression of blood vessel occlusion

**Figure 2:**
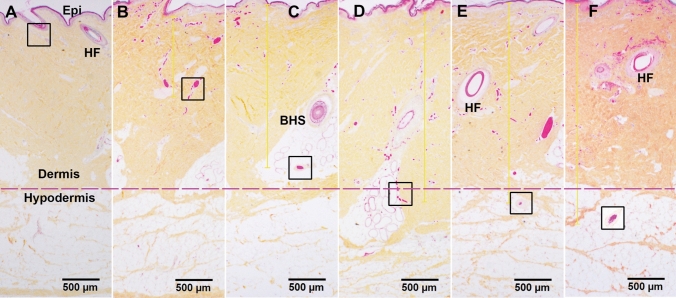
Representative HPS micrographs of burn site of porcine burn wounds harvested at 0 h (A), 0.5 h (B), 1 h (C), 2 h (D), 4 h (E), and 24 h (F) after injury. The box in each time point pinpoints the deepest vertical (from epidermal basement) levels of vessel occlusion at that time point. Higher power images of these boxes are presented in Fig. 3. Epi, epithelium; HF, hair follicle; BHS, the base of the hair shaft. Bar = 500 µm (A to F).

**Figure 3:**
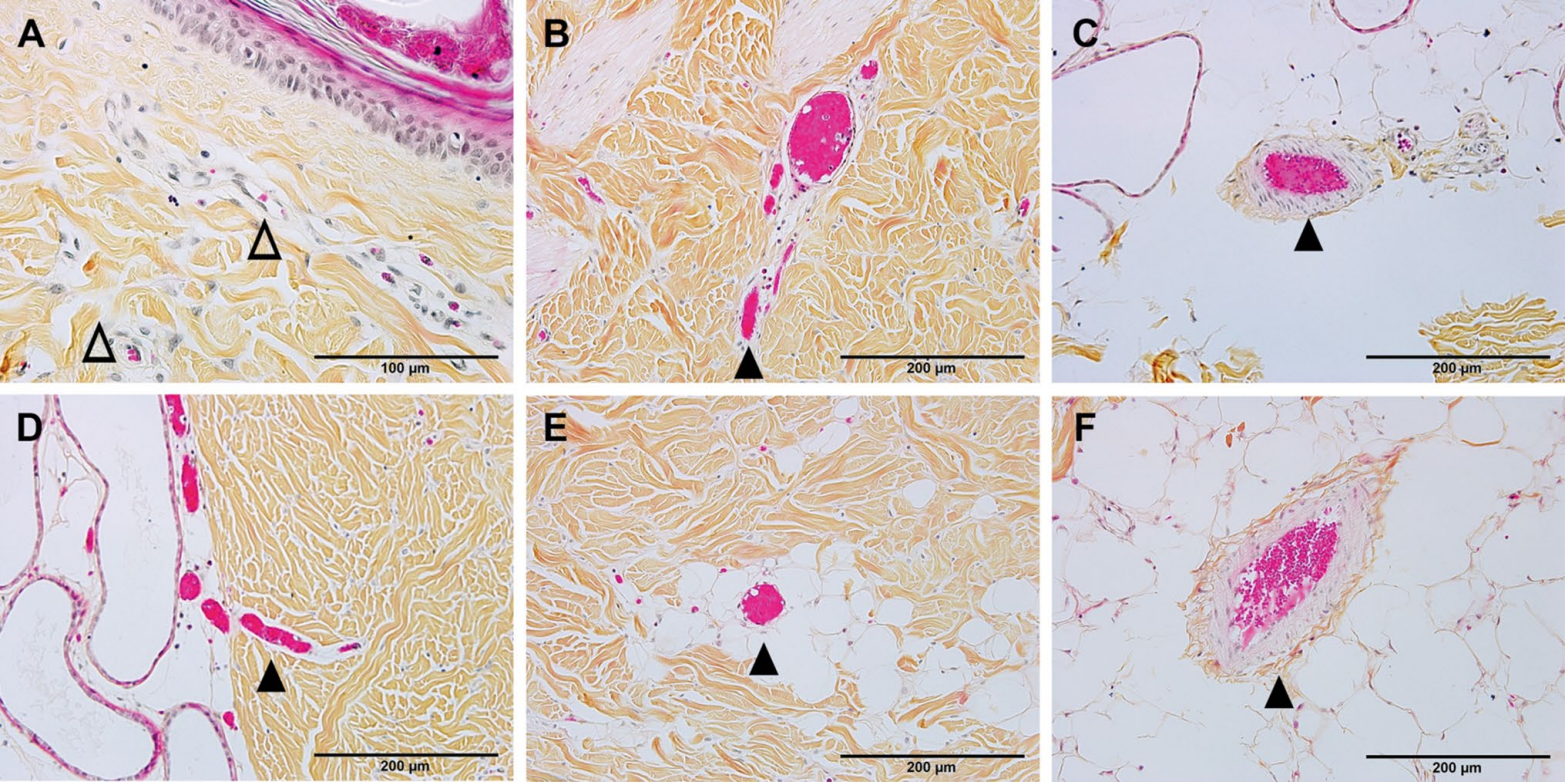
**A**, **B**, **C**, **D**, **E**, and **F** are high-power images of the boxes outlined in the corresponding micrographs in Fig. 2. These photomicrographs are taken from the burn site of porcine burn wounds harvested at 0, 0.5, 1, 2, 4, and 24 h, respectively. ∆, patent vessels beneath the epidermis of control. ▲, blocked blood vessels. Bar = 200 µm (**B** to **F**), 100 µm (**A**).

As illustrated in Fig. 2, the depth of blood vessel occlusion increased or progressed with time. Blood vessel occlusion was seen by the middle of the dermis at 30 min down to the bottom of the dermis at 2 h, and then down to the upper portion of hypodermis by 4 to 24 h after burn. Figure 3A–F is the higher power of the boxes in the corresponding micrographs in Fig. 2. The high magnification images in Fig. 3 further confirmed the presence of clear small blood vessels (∆) beneath the epidermis at 0 h (control), and full blood vessel occlusion in the middle of the dermis, the bottom of the dermis as well as the upper portion of hypodermis in the burn sites as time progress.

**Figure 4:**
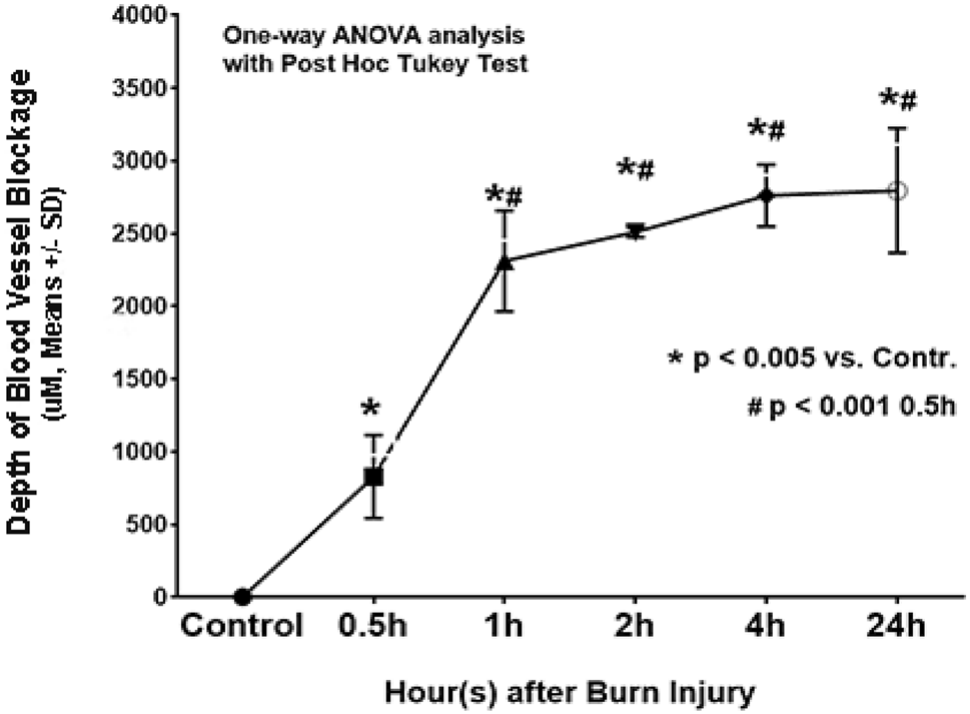
A diagram showing the progression of the depth of blood vessel occlusion measured as the distance from the epidermis to the deepest vertical (from the epidermal basement) location of blood vessel blockage. The measurements were performed on the burn sites of porcine burn wounds harvested at various time points after injury.

Measurements of the depth of blood vessel occlusion in the burn sites showed increased progression of the brass comb burn wound (Table 1). This depth increased at 30 min (p < 0.005 vs control), reached a significantly higher level at 1 h after burn (p < 0.0001) that continued to increase up to 2 h (p < 0.005). The depth of blood vessel occlusion increased slightly from 2 to 4 h then to 24 h, but these increases were not significant (p > 0.05) compared to the values measured at 1 h or 2 h. Graphical representation of the depth of blood vessel occlusion, depicted in Fig. 4, shows a 3-phases progression: 1- an immediate response within 30 min, 2- a rapid progression from 0.5 h to 2 h, and 3- a slow progression from 2 to 24 h after the initial injury (Figs. 2, 3, and 4).

**Table 1:** Depth of blood vessel occlusion and endothelial cell death.

Measurement^a^	Hours after brass comb burn injury					
	0^b^	0.5	1	2	4	24
Depth of blood vessel occlusion	0 ± 0	0.83 ± 0.29*	2.31 ± 0.35*#	2.51 ± 0.03*#	2.76 ± 0.21*#	2.80 ± 0.43*#
Depth of endothelial cell death^d^	0 ± 0	1.19 ± 0.31*	1.11 ± 0.24*	1.15 ± 0.11*	1.85 ± 0.34*^#$^	2.92 ± 0.62*^#$&^

^a^millimeters, ^b^ setting up as controls (normal pigskin with no brass comb burn; ^c^ defined as the deepest vertical (from epidermal basement) location of visibly dilated venules and/or arterioles filled with denatured clots on burn sites of HPS trichrome stained skin wound sections; ^d^ defined as the deepest vertical (from epidermal basement) location of positively labeled blood vessels on burn sites of vimentin-IHC stained skin wound sections.

*p < 0.005–0.0001, versus corresponding controls (0 h); ^#^ *p* < 0.003–0.0001, versus corresponding 0.5 h; ^$^ *p* < 0.003–0.0001, versus corresponding 1 h and 2 h; ^&^ *p* < 0.03, versus 4 h.

### Endothelial cell death

#### Histochemistry features

**Figure 5:**
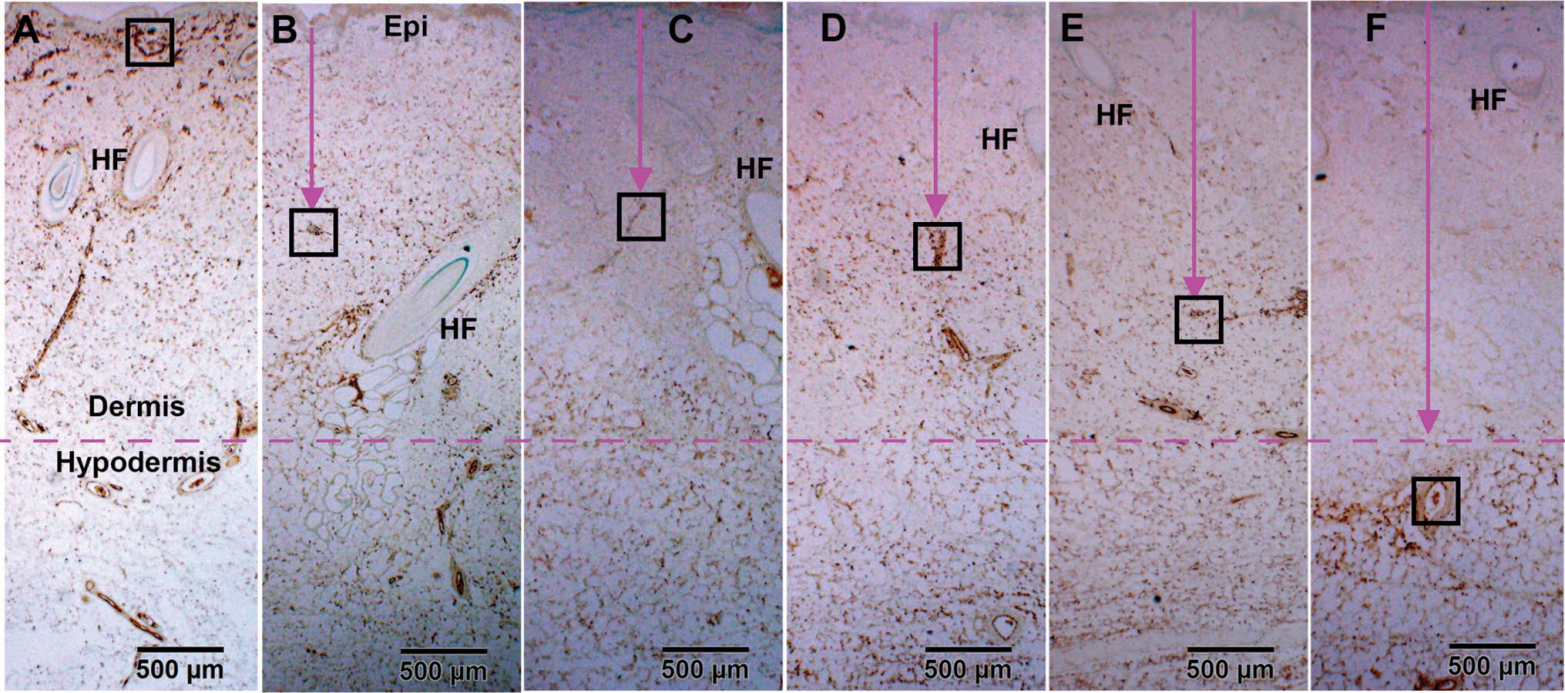
Representative vimentin IHC micrographs of burn sites of porcine burn wounds harvested at 0 h (**A**), 0.5 h (**B**), 1 h (**C**), 2 h (**D**), 4 h (**E**), 24 h (**F**). The box in each time point was outlined to locate the highest vertical (from epidermal basement) levels of positive vimentin staining at that time point. High power images of the outlined boxes are presented in Fig. 6. Epi, epithelium. HF, hair follicle. Bar = 500 µm (**A** to **F**).

**Figure 6:**
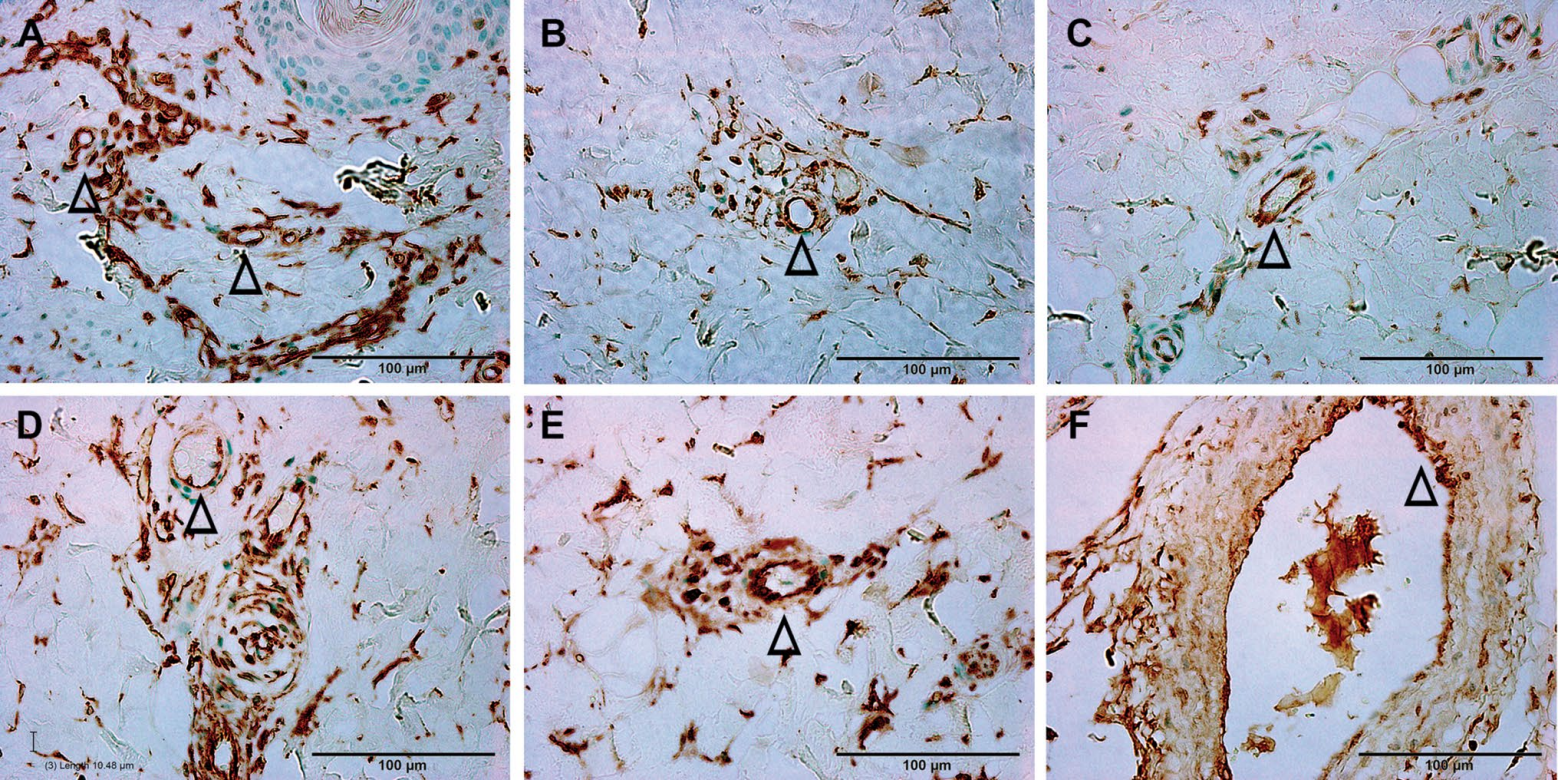
High power images of vimentin IHC shown in the boxes outlined in the corresponding micrographs in Fig. 5 and representing the burn sites of porcine burn wounds harvested at 0 h (**A**), 0.5 h (**B**), 1 h (**C**), 2 h (**D**), 4 h (**E**) and 24 h (**F**), respectively. ∆, patent blood vessels Bar = 100 µm (**A** to **F**).

Analysis of control skin and unburned interspaces of the burn wounds shows a strong positive vimentin IHC-P staining of uninjured blood vessels from the upper dermis (just beneath the basement of the epidermis, Figs. 5A and 6A) to the hypodermis (Fig. 5A). In the burn sites, clear negative labeling of blood vessels was observed from the dermis to the hypodermis depending on the time point of sample collection (Figs. 5B–F and 6B–F), making vimentin IHC-P a useful marker to monitor the progression of endothelial cell death after-burn. Although negative vimentin-staining of blood vessels is not direct evidence of endothelial cell death, the negative staining means that the specific antibody did not recognize the binding structures of the antigen due to structural changes such as degeneration, or disorganization, which generally means irreversible structure change or cell death. Hematoxylin and eosin (H&E) stained sections from the burn sites showed increased nuclear condensation in the epithelial and endothelial cells, indicative of dead cells. Nuclear condensation was not observed in the interspace of the same section, 1 h after thermal burn (100 °C for 30 s) (data not shown).

#### Progression of endothelial cell death

**Figure 7:**
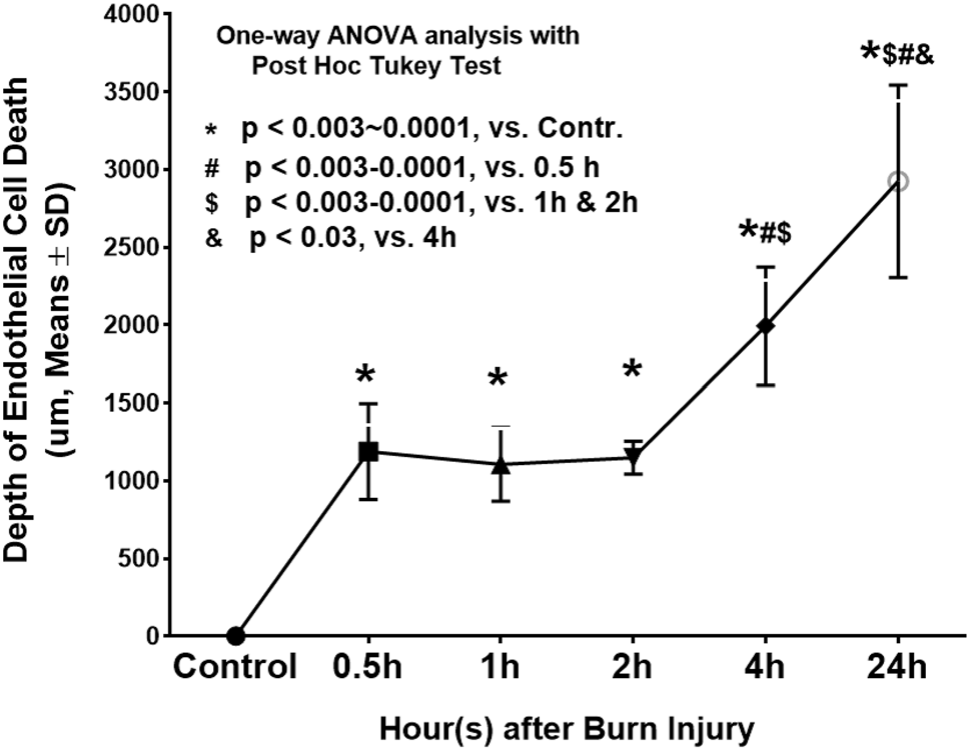
A diagram showing the vertical progression of endothelial cell death measured as the distance from the epidermis to the highest vertical location of positively vimentin-stained endothelial cells on the burn site of porcine skin biopsies harvested at various time points after burn.

As shown in Figs. 5 and 6 and quantified in Table 1, the depth of endothelial cell death increased significantly by 30 min after burn (p < 0.005 vs control) and stayed at the middle dermis through 2 h. Endothelial cell death continued to increase significantly at 4 h (p < 0.003 vs 0.5, 1, and 2 h), and 24 h (p < 0.03 vs 4 h). Figures 5 and 6 demonstrate the temporal progressive vertical loss vimentin-staining (endothelial cell death), from the middle of the dermis at 30 min down to the bottom of the dermis at 4 h, and then down to the upper portion of hypodermis at 24 h. The temporal progression of the loss of vimentin-positive cells indicative of vascular endothelial cell death also shows 3 phases: an immediate response within 30 min, a plateau phase from 30 min to 2 h, and a marked progression from 2 to 24 h (Fig. 7).

## Discussion

Previous studies have documented histological time courses of burn injury progression in animal models. For instance, histology changes of burn wounds were recorded at 24 h only^11–13^. Dilated capillaries in hypodermis on H&E-stained sections were observed at 4 h after burn^15^. Histological alterations of burn marks (including vascular) on H&E-stained skin samples were reported at 2, 24, 48, and 72 h^24^. The depth of injury in the burn site and dermal components including collagen, hair follicles, endothelial cells, and interstitial cells were observed at 1, 24, 48 h, 7 days, and 28 days after injury^25^. Endothelial cell death was seen as early as 1 h after burn^25,26^. The current study provides a morphological time-course of blood vessel occlusion and vascular endothelial cell death as early as half-hour after burn. As expected, the current study demonstrates an early vertical progression of blood vessel occlusion and vascular endothelial cell death in the burn sites of the porcine model of brass comb burn with wider interspace (10 mm rather than the 5 mm interspace used by Regas and others)^11–13,25,27^. The time course of the vertical blood vessel occlusion showed 3 phases of progression: an immediate response from the epidermis to the upper dermis within 30 min after the initial injury, a rapid progression phase from the upper dermis to the bottom of the dermis between 0.5 h and 2 h, and a slow progression from the lower dermis to the hypodermis between 2 and 24 h (Figs. 2 and 3). The vertical progression of vascular endothelial cell death also showed three different phases: an immediate response down to the middle of the dermis within 30 min, a plateau phase at the same depth from 30 min to 2 h, and a marked progression from 2 to 24 h (Figs. 5 and 6). The immediate response might be a direct impact of heat energy, to which there may be no choice to deal with except cooling measures to reduce heat energy itself. However, the rapid progression after the 30 min increase should be the result of a secondary injury derived from the heat injury. The time window before and during the rapid progression phase may be the optimal time window for the application of therapeutic interventions to get the best outcomes. Blood vessel occlusion has a window of 2 h. after burn and vascular endothelial cell death has a window of 4 h. In addition to the optimal time window, the first 24 h after initial thermal insult is also suitable for therapeutic intervention since both pathological entities progressed to hypodermis 24 h after burn. These results add morphological evidence of early vertical progression of burn wounds and define the optimal time window for therapeutic interventions to reduce or prevent injury progression.

These data presented the difference in the progression time window between blood vessel occlusion and vascular endothelial cell death (Fig. 4 vs Fig. 7). The blood vessel occlusion showed a rapid and short vertical progression within 2 h, which is before a vertical progression window of vascular endothelial cell death within 4 h. While the mechanism underlying the difference and the relationship between the two pathological entities are not clear, our data indicate that blood flow reduction precedes vascular endothelial cell death. This result is supported by a previous study showing that a three times pretreatment with subcutaneous injection of heparin (8 h. apart) preserved vascular integrity up to 72 h after injury^9^. However, other studies proposed other players such as inflammatory factors as contributors to vascular endothelial cell death^23,28^.

In the burn sites, blood vessel occlusion is the morphological presentation of blood flow cessation. The occlusion may be divided into two categories: initial and advanced. The initial occlusion occurs immediately after the contact of heat energy with the skin surface. The depth of initial blood vessel occlusion may spread from the top superficial portion of the upper dermis to the deep bottom of the hypodermis and even to the muscle layer and beneath structures. The extent of the damage depends on the level of heat energy released to the skin and/or the duration of the contact of heat with the skin. As we know, the heat energy might be exhausted within a few seconds or minutes. The initial occlusion, however, and accompanying anoxia/ischemia might be persistent due to the direct impact of heat energy. Therefore, the initially occluded vessel does not have the opportunity of reperfusion. The initial occlusion usually progresses down to deeper skin layers with time as demonstrated in this report. The time window that initial occlusion progresses from the upper dermis down to the hypodermis provides the opportunity for therapeutic interventions to minimize or prevent burn wound progression. This time window is within 2 h to 4 h after injury based on this report’s results.

The causative factors of blood vessel occlusion and endothelial cell death are not defined clearly, and the mechanisms underlying these pathological entities are complicated^29^. Although the current study did not focus on the mechanisms underlying the development of these pathological changes, the presented results are in line with the previous findings^29^: heat energy is a major factor responsible for the early injury progression within 1 to 2 h after initial thermal exposure. RBC aggregation or microthrombi on the superficial layer of the dermis are a direct insult of heat energy. RBC aggregates may result in blood flow reduction and cessation early after injury, but other factors may also contribute to the early blood flow cessation. For example, direct injury of heat energy to capillaries may also induce blood flow reduction and cessation. The impact of heat energy induces a contraction of arterioles in the superficial dermis leading to a reduction in blood flow. The obstruction of arterioles, capillaries, and venules causes injury progression during the late phases. Other contributing factors including, but are not limited to, the contraction of arterioles and other small arteries, pressure from stiff skin, increased vascular permeability-induced to interstitial edema, and inflammatory factors.

The progression time windows of blood vessel occlusion (blood flow cessation) in burn sites (zone of coagulation) of the porcine brass comb burn model are different from some previous reports. Regas and Ehrlich^11^, for instance, used a laser Doppler flowmeter to monitor blood flow near the surface of normal control skin, burn sites, and unburned interspaces. They reported a progressive decrease in blood flow in the untreated interspace, but not in untreated burn sites for the first 24 h after injury^11^. Decreased blood flow was observed in the zone of stasis (interspace) and zone of coagulation at 5 min, 1, 2, 4, 8, 12, and 24 h after injury^12^. The same study reported a progressive decrease of blood flow in the zone of stasis and an immediate and decrease in blood flow in the burned area (zone of coagulation) from 5 min to 24 h after injury, with no significant differences observed from 1 to 24 h^12^. Blood flow in the burn sites (zone of coagulation) measured with a laser Doppler flowmeter progressively decreased from 50% of control at 1 min to about 15% of control by 5 min after injury, and stayed the same level for 2 h thereafter^13^. The difference between this study and our report may be due to the difference in contact burn probe used; the current study used a brass comb with wider interspaces (10 mm) compared to the 5 mm interspaces used other studies of. The narrow (5 mm) notches may accumulate more heat energy radiating from both teethes compared to the wider notches such as 10 mm^19^, which may induce a false injury progression not from the zone of coagulation but partially from heat energy. Whether the narrow notch makes the difference needs further clarification.

Our results are in line with previous studies. Assessment of dermal ischemia using postmortem arteriography of radiopaque dye in a rat model of full-thickness burn covering 20% of total body surface (TBSA) showed vascular compromise between 2 and 4 h progressing to complete de-vascularization by 24 h after injury^9^. Vo et al. employed fiber-optic confocal imaging (FOCI) and laser Doppler flowmetry to detect changes in vascular morphology and local blood flux on hairless mice at 1, 2, 3, and 4 h after burn^15^. They found that blood flux in the zone of coagulation decreased significantly (p < 0.01 to 0.005) each hour during a 4-h period^15^. No significant changes in blood flow were observed in the zone of stasis during the same period, but increased blood flow was observed in the zone of hyperemia at 1, 2, and 3 h after injury^15^. Papp et al.^24^ analyzed the progression of burn depth in the ventral torso of a pig after exposure to a heated block for 1, 3, 6, 9, or 12, and found that, except for the 1-s exposure, all times of heat exposure induced injury from middle dermis to the deep dermis when examined at 2 h. Injury progression to the hypodermis was evident at 24 h for the 6, 9, and 12-s heat exposure. Heat exposure for 3 s did not show progression to hypodermis until 48 h after burn^24^. The current study does not only document early histological time-course of vertical burn injury progression in the burn sites (zone of coagulation) but also provides morphological evidence to support other findings of early blood flow changes measured by laser Doppler flowmeters.

The results presented here have limitations. Brass comb burn induces contact burn injury on a small skin island, 5.7 cm^2^ per comb, about 0.09% of TBSA. For a maximum of 20 comb burn sites on one pig, the total burn size is about 1.8% TBSA; a very small burn size when compared to clinical burns, such as 50% of TBSA burn. Therefore, the mechanisms underlying burn injury progression in humans may be more complicated since more factors are involved in large burns, such as impairment of the immune system, infections, and insufficient systemic perfusion. Additionally, the brass comb contact burn may be more damaging than the other burns such as scald burn, since the metal may transmit heat energy faster and/or longer.

## Conclusions

In summary, the current study demonstrated morphological evidence of blood vessel occlusion and endothelial cell death as early as 30 min after contact burn in a porcine model and reveals morphological time-course of burn progression in this model. We observed vertical progression of both blood vessel occlusion and vascular endothelial cell death from the middle of dermis down to hypodermis within 2 to 4 h after the initial contact burn, namely a progression from secondary degree (partial thickness) to third-degree (full thickness) burns. More importantly, this report shows the time windows in which efficacious therapeutic interventions may minimize or prevent burn wound progression. These data encourage further investigations to define the time course for other pathologic markers of burn wound progression such as the death of epithelial and mesenchymal cells, collagen degeneration, and cessation of hair follicle stem generation. Since blood flow cessation is essential in burn progression, understanding the mechanisms underlying blood vessel occlusion is of paramount importance to develop therapeutic interventions.

## Materials and methods

### Materials

The brass comb used in this study was a modified version of the Regas and Ehrlich^11^ model and was used previously in our rat studies^19^. The modified mold produces three 10 × 19 mm rectangles of burn sites separated by two 10 × 19 mm rectangles of unburned interspaces.

Hematoxylin phloxin saffron (HPS) kits (cat. #k023) for HPS staining were purchased from Poly Scientific R&D Corp. (Bay Shore, NY). Mouse monoclonal anti-vimentin antibody (V9) was obtained from Sigma (cat#347 M-18, Cell Marque, St Louis, MO).

### Animals

A total of 3 Class A 3-month-old Yorkshire pigs (30–31.8 kg) received a brass comb contact burn in this study. Two pigs were studied for 4 h after contact burn under surgical anesthesia and analgesia, during which burn wounds were sampled at 0.5, 1, 2, and 4 h. Unburned skin was only collected at 4 h. Once awake from anesthesia, the third pig was placed in a sling to be monitored for up to 24 h after the initial injury. Skin biopsies were harvested from burn and unburned areas at the end of the study under anesthesia. All three animals were sacrificed under anesthesia after skin tissue sampling. The animals were given a standard porcine diet (LabDiet 5084, PMI Nutrition, IN) and water ad libitum. The Institutional Animal Care and Use Committee of the University of Texas Medical Branch at Galveston approved all animal manipulations. Housing and care of the pigs met the National Research Council guidelines. All animal studies were carried out following the ARRIVE guidelines.

### Animal experimental protocol

After premedication with an intramuscular injection of ketamine and xylazine and inhalation anesthesia with 2–3% of isoflurane in room air via facemask, pigs were weighed and the dorsum of each pig was completely clipped with an electrical clipper in a separate preparation room. The animals were then transferred to the operating room, placed on an operating table in a prone position, pre-oxygenated with 40% oxygen via inhalation mask. The pigs were intubated and inhalation anesthesia delivered by isoflurane 1–3% for the whole experimental period. A venous line was established in an ear vein and infusion of Ringer’s lactate solution continued for the entire length of the experiment. The animal’s dorsum was marked on each side with two lines of 19 × 50 mm rectangles, resulting in a subtotal of 9 rectangles of 19 × 50 mm on each side between the caudal end of the scapula and rostral end of the ilium and, a total of 18 rectangles of 19 × 50 mm per animal. Each rectangle in line was about 20 mm apart from the other. The line near the middle was parallel to and about 10 mm distal to the middle line. The distance between the two lines on each side was 20 mm apart. For the animals that survived for 4 h after burn, adjacent outlined rectangles were randomly divided into the following groups: control (n = 2 sites), without burn; Burn alone (100 °C for 30 s) at 0.5, 1, 2, and 4 h (n = 4 sites for each time point). For the animal that survived for 24 h, a total of 8 outlined rectangles were randomly distributed equally on each side of its dorsum and divided into the following groups: control not burned (n = 3 sites); Burn alone (100 °C for 30 s) at 24 h (n = 8 sites). The designed control rectangles received no insult. The burn-alone rectangles received contact burn only and the wounds were exposed without any covering.

The contact thermal injury was induced by applying a pre-heated brass comb for 30 s, on each pre-marked rectangle, with no additional pressure. The comb weighs 316 g and was pre-heated in boiling water (100 °C) for 3 min. For pre-and postoperative analgesia, transdermal fentanyl patches (100 mcg/h) (Duragesic 100, Janssen Pharmaceutics, NJ) were applied on the dorsal neck behind the ear 24 h pre-procedures and kept in place by using surgical staples and covered with an adhesive transparent dressing. For additional analgesia, Buprenorphine (0.01–0.05 mg/kg IM) or Buprenorphine SR (0.12–0.3 mg/kg SC) was given if needed.

The samples were grouped in a total of 6 sample groups/time points were analyzed: 1 control group (0 h) and 5 burn groups (0.5, 1, 2, 4, and 24 h). Four (4) burn wounds or control skin samples were randomly picked from each group of samples harvested from burn wounds and unburned skin at different time points, yielding a total of 24 (4 × 6) samples.

### Laboratory procedures

#### HPS staining

To identify blood vessel occlusion, HPS staining was carried out as described previously^19^: 24 frozen sections from 24 formalin-fixed paraffin-embedded skin samples were de-paraffinized, rehydrated, and stained per manufacturer instruction with the HPS trichrome kit.

#### Vimentin immunohistochemistry

To identify endothelial cell death in the blood vessels, we performed vimentin immunohistochemistry. Frozen sections from 24 formalin-fixed paraffin-embedded skin samples were deparaffinized, rehydrated, and treated with citrate buffer before washing with 0.01 M phosphate-buffered saline (PBS). The sections were then quenched for 10 min in 3% hydrogen peroxide/methanol, washed with PBS again, blocked for 1 h with blocking solution (5% normal goat serum/2% BSA/0.1% cold fish skin gelatin/ 0.1% Triton X-100/0.05%Tween 20/0.05% sodium azide in 0.01 M PBS). The sections were washed again, blocked with avidin/biotin (15 min each with brief PBS wash between), incubated overnight at 4 °C with mouse monoclonal anti-vimentin antibody (V9) from Sigma (cat#347 M-18). After PBS washing, the sections were incubated for 90 min with a secondary biotinylated goat-anti-mouse antibody, washed, and processed in the dark for 1 h with the avidin–biotin complex reagent (standard Vectastain ABC Elite Kit; Vector Laboratories, Burlingame, CA). After washing with PBS, the sections were developed with a mixture of DAB substrate (3,3′- Diaminobenzidine) (Dako, Carpinteria, CA) followed by counterstaining with 0.5% methyl green. Determination of the optimal concentration of primary antibody and negative control was carried out at the beginning of the study. Negative control staining was performed by omitting the primary antibody step.

#### Morphometric measurement and data collecting

All skin sections of HPS trichrome staining and vimentin immunohistochemistry (IHC-P) staining were visualized using an Olympus BX53 digital microscope. Images were acquired using the Olympus CellSense program. Blood vessel occlusion and endothelial cell death were viewed under different objectives (4x, or 10x, or 20x) to ensure the pathological changes. The depth of blood vessel occlusion and vascular endothelial cell death was measured in micrometers (µm) under a lower power objective (2x) with the aid of the Olympus CellSense software.

The depth of the blood vessel blockade was defined as the deepest vertical (from epidermal basement) location of visibly dilated venules and/or arterioles filled fully with denatured clots on burn sites of HPS trichrome stained sections. Negative vimentin staining is suggestive of blood vessel damage or endothelial cell death and ideally used to monitor endothelial cell death. However, it is not feasible to identify negative-stained blood vessels because of the lack of staining. Therefore, the depth of endothelial cell death was defined as the vertical distance from the epidermis to the highest location of positively in vimentin-stained vascular cavities in the middle of burn sites.

Since each sample was trimmed into 3 identical segments (each has 2 burn sites), there were 6 burn sites per section, 24 sites (6 × 4) per group, and a total of 144 burn sites (6 × 4 × 6) per staining, yielding a total of 288 (144 × 2) measurements. Two operators carried the microscopic measurements, and one of them was blinded to the group designation. Each microscopic measurement was scored and recorded when the two operators agreed on the score.

### Data analysis

Measurement of the depth of blood vessel occlusion and endothelial cell death on both burn sites for each section resulted in six readings for each comb wound, which were then averaged to one value to represent a single wound. The average values of four wound samples in each group were statistically analyzed and expressed as Mean ± SD. One-way ANOVA in conjunction with Tukey’s post hoc analysis was used to stratify and determine differences between the groups by using Prism from GraphPad (San Diego, CA). Differences were considered significant at p < 0.05.
